# Ventilatory efficiency (η⩒E) of the exercise: A detailed method report

**DOI:** 10.1016/j.mex.2023.102412

**Published:** 2023-10-05

**Authors:** Paulo de Tarso Muller

**Affiliations:** Federal University of Mato Grosso do Sul (UFMS)/Maria Aparecida Pedrossian Hospital (HUMAP), Laboratory of Respiratory Pathophysiology (LAFIR), Campo Grande MS, Mato Grosso do Sul, Brazil

**Keywords:** Ventilation, Efficiency, Exercise, Ventilatory Efficiency Index

## Abstract

Ventilatory efficiency is a combination of the ventilatory-metabolic response stemming from non-invasive analysis of cardiopulmonary exercise testing. Despite being a recognized marker in exercise physiology, this measure presents considerable limitations, including the imprecise designation of “efficiency”, broadly recognized, and recently denominated as “excess ventilation”. Herein we present a detailed method, with substantial improvements, and new physiological insights, in order to better define the true ventilatory efficiency of the exercise, according to recommendations for physical/physiological processes.

• “Ventilatory efficiency” of the exercise is a remarkable physiological index.
• Several limitations are currently debated.
• We report a new ventilatory efficiency index that match with recommendations.

## Graphical abstract

Example of ventilatory efficiency (η^⩒^_E_) calculation. In this three-axis plot, minute-ventilation was plotted without *log* scale and placed at the right y-axis, for clarity. The linear part of the quadratic function is depicted as the inclined red line, calculated as the coefficient or actual CO_2_ output constant rate, or ⩒CO_2_-log⩒_E_ slope = 3 L_*_ logL_ˉ_¹, for this particular participant. The maximal predicted ventilatory ventilation (MVV) for this participant is 140 L_*_min^−1^, and the maximum “assumed” CO_2_ output constant rate (⩒CO_2_-log⩒_E_ max) theoretically predicted would be 26 L_*_ logL_ˉ_¹ (see the text for elaboration). Thus, η^⩒^*_E_ = (*⩒CO_2_-log⩒_E_ slope / ⩒CO_2_-log⩒_E_ max) * 100 = 11,5 %. Reproduced with permission.

**Figure:**
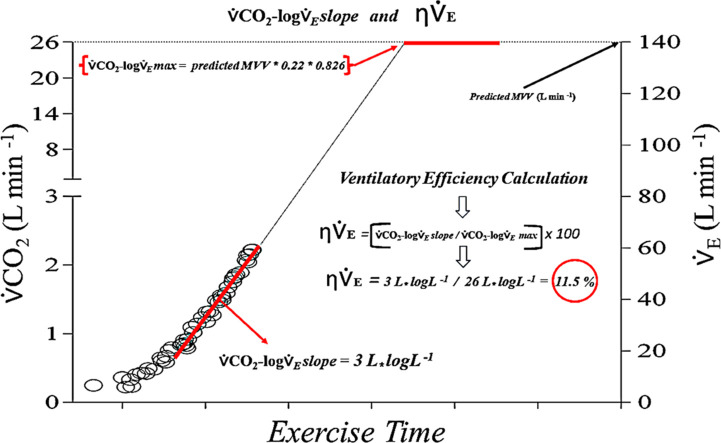

Specifications Table

**Table:** 

Subject area:	Medicine and Dentistry
More specific subject area:	Respiratory Physiology
Name of your method:	Ventilatory Efficiency Index
Name and reference of original method:	1. Ventilatory Efficiency Index described previously by the author for validation in chronic obstructive pulmonary disease participants. The work was published as follow: P. T. Muller, E. F. Saraiva, Ventilatory inefficiency during graded exercise in COPD: A pragmatic approach, Clin. Physiol. Funct. Imaging. 41 (2021) 1:103–109, doi:10.1111/cpf.12674. 2. Ventilatory Efficiency Index described previously by the author for validation in smokers without COPD. The work was published as follow: P. T. Muller, G. G. Orro, G. W. Barbosa, E. Saraiva, A new ventilatory efficiency index and accuracy for early lung diffusion impairment in non-COPD smokers, Respir. Physiol. Neurobiol. 289 (2021) 103,670, doi:10.1016/j.resp.2021.103670.
Resource availability:	1. Ventilatory Efficiency Index described previously by the author for validation in chronic obstructive pulmonary disease participants. The work was published as follow: P. T. Muller, E. F. Saraiva, Ventilatory inefficiency during graded exercise in COPD: A pragmatic approach, Clin. Physiol. Funct. Imaging. 41 (2021) 1:103–109, doi:10.1111/cpf.12674. 2. Ventilatory Efficiency Index described previously by the author for validation in smokers without COPD. The work was published as follow: P. T. Muller, G. G. Orro, G. W. Barbosa, E. Saraiva, A new ventilatory efficiency index and accuracy for early lung diffusion impairment in non-COPD smokers, Respir. Physiol. Neurobiol. 289 (2021) 103,670, doi:10.1016/j.resp.2021.103670.

## Method details

### Rational

The relationship between minute ventilation (⩒_E_) and carbon dioxide output (⩒CO_2_) is a non-invasive physiological mutual response to exercise that is influenced by several factors (e.g., chemosensitivity, ergoreceptors, lung perfusion, etc.), and finely coupled to exercise demands [1]. The ⩒_E-_⩒CO_2_ slope is a classic marker of “ventilatory efficiency” [2], and there is large agreement of its importance in clinical settings [2], [3], [4]. However, there are several points that remain inconclusive with respect to the reliability of the ⩒_E-_⩒CO_2_ slope. Thus, among the limitations of the ⩒_E-_⩒CO_2_ slope are (i) the recognized effects of ventilatory mechanical constraints in down-shifting the ⩒_E-_⩒CO_2_ slope [5,6], (ii) the imprecise use of the designation “ventilatory efficiency” with respect to the ⩒_E-_⩒CO_2_ slope [7,8], (iii) the imprecise application of linear regression for a quadratic function, that fits better when using the whole exercise time-frame, including the post respiratory compensation point (RCP) data [1], (iv) the use of a smaller sample of data points for the linear segment of the ⩒_E-_⩒CO_2_ response, excluding data on remarkable physiological ventilatory responses after the RCP [9], and (v) the effects of primary hyperventilation [7]. In addition, (vi) severe acidosis could up-shift the ⩒_E-_⩒CO_2_ slope at the level comparable with cardiopulmonary disease (slope > 35) in athletes [10], and (vii) recently, several limitations of the ⩒_E-_⩒CO_2_ slope for endpoint prognostication in cardiopulmonary diseases were described [11,12]. The method presented herein is an alternative to the ⩒_E-_⩒CO_2_ slope, and the first effort at a detailed description of ventilatory efficiency of the exercise (η^⩒^_E_), that is consistent with the true designation of “efficiency” in biology/physiology [13], characterized by the following criteria: (i) *“efficiency is a measure of performance that characterizes an actual performance relative to a perfect level”*, (ii) and “*must be preferentially presented as a fraction (percentage) of a single process”*. This paper substantially improves the method previously published by the same author for validation [6,14].

### Exercise protocol and data analysis

The CPET was performed in a Vmax *encore* 229® device (Viasys Healthcare, Yorba Linda, USA, 2012), controlled by an electric braked cycle ergometer ViaSprint (Viasys Healthcare, Yorba Linda, USA, 2012), under digital oxygen saturation and electrocardiography monitoring (Cardiosoft®, EUA, 2012). Subjects with HF exercised through an incremental protocol to a maximal symptom-limited performance with a 10–20 Watt_*_min^−1^. Healthy subjects exercised with 25 Watt_*_min^−1^. Details of the method were published previously [15,16]. The individual tests of the exercise phase were analyzed breath-by-breath, with exclusion of values exceeding the standard deviation of the local average by 3-times, before presenting five-breath moving averages for ⩒CO_2_-log⩒_E_ slope and η^⩒^_E_ analysis and the method description.

## Method description

### ⩒CO_2_-log⩒*_E_* slope

**Fig. 1:**
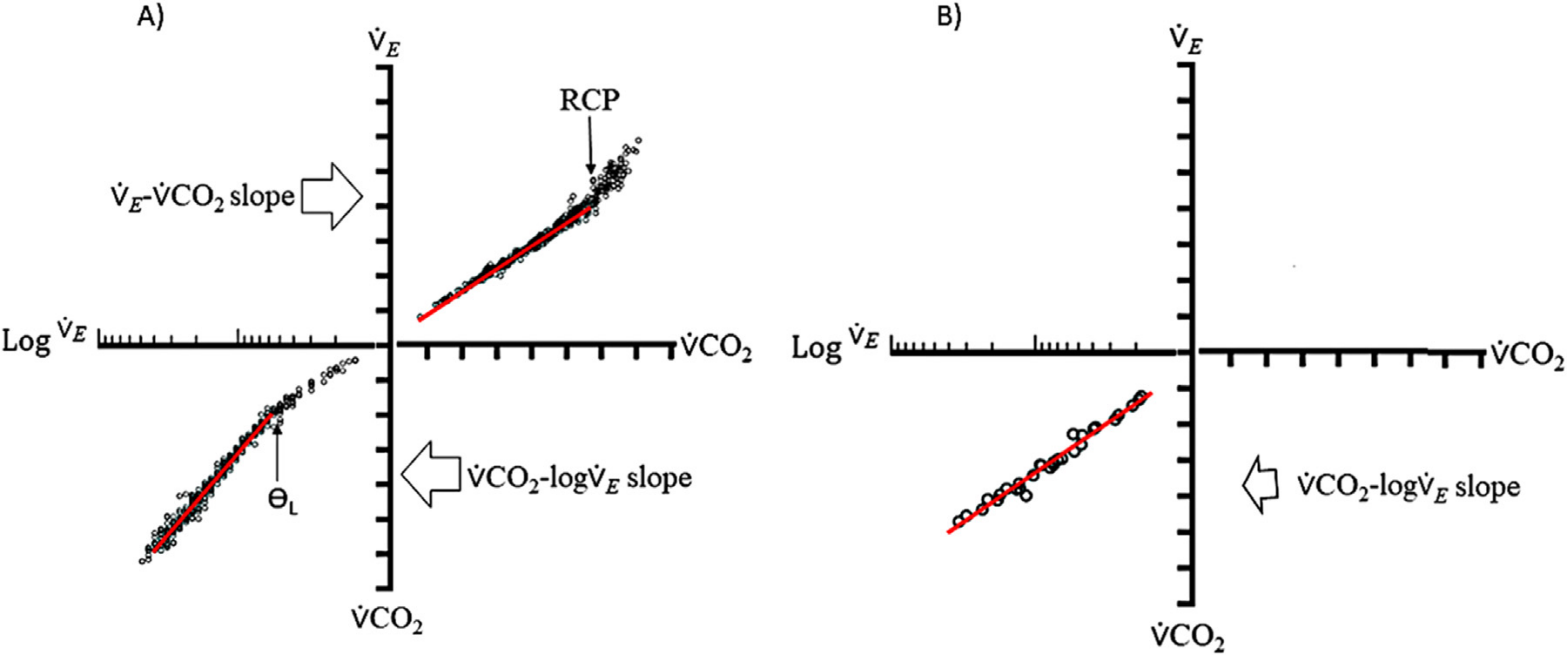
Integrated four-axis representation for ventilatory efficiency. We can see in the right upper axis the quadratic function response for the ⩒*_E_*-⩒CO_2_ slope from the start-to-end of exercise. The “red line” comprises the pre-RCP regression function (A). In the left lower is depicted the quadratic function for the ⩒CO_2_-log⩒*_E_* slope from the start-to-end of exercise response. The “red line” comprises the linear part of the total response (A and B). The left x-axis is represented as log⩒E. Abbreviation: RCP, Respiratory Compensation Point.

The method is based on measuring the amount of CO_2_ cleared by the lungs (⩒CO_2_, L_*_min^−1^) plotted against a predefined range of increase in minute-ventilation (⩒_E_) (ten-fold increase, based on *semi-log* scale) during incremental exercise to symptom-limited maximum tolerance. During this *semi-log* construction, in most cases the ⩒CO_2_-log⩒_E_ slope stemmed from a quadratic function, with part of the equation better fitted by a true linear function (red line in Fig. 1A, bottom left). In some individuals, data might present a linear tendency, from the beginning, up to end of the exercise (Fig. 1B). Thus, the CO*_2_* output is represented as a dependent variable (y-axis), and ⩒_E_ as the independent variable (x-axis). To achieve a constant rate of CO*_2_* output, we took the log*_10_* of the ⩒_E_, as previously described for the oxygen uptake efficiency slope (OUES). During this *semi-log* plotting, the ⩒CO_2_-log⩒_E_ signal is described by a quadratic function, as follow:

(1) $$\begin{array}{c} \dot{\vee }CO_{2}=a_{*}{\dot{\vee }}_{E}^{2}+{\dot{\vee }}_{E}+c \end{array}$$

with the final component of the equation described by a linear function:

(2) $$\left(b_{*}{\dot{\vee }}_{E}+c\right)$$

**Fig. 2:**
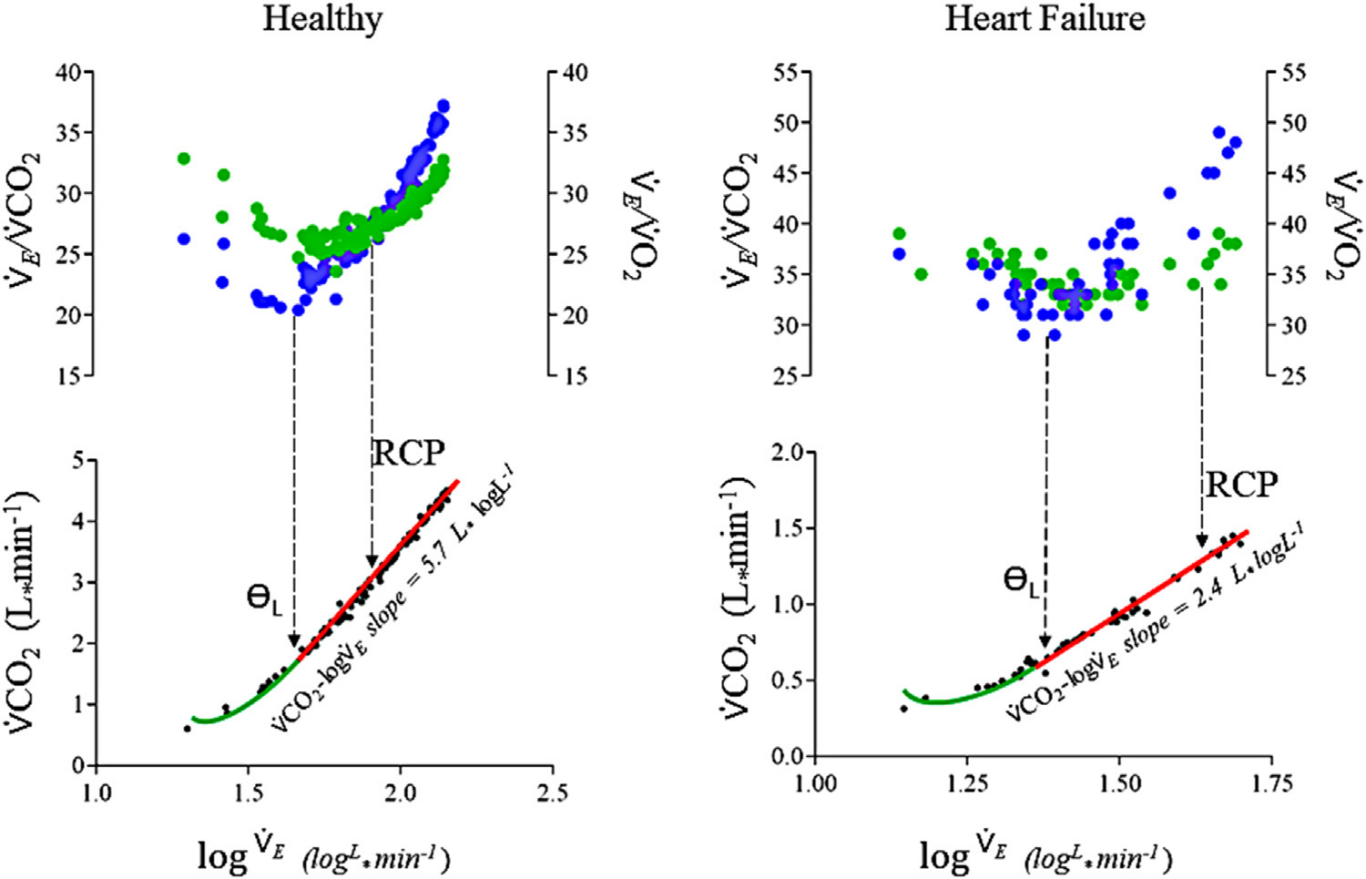
Representation of the ⩒*_E_*/⩒CO_2_ and ⩒*_E_*/⩒O_2’_ equivalents against log⩒*E* common x-axis in the Upper Panel, and ⩒CO_2_ (L) against log⩒*E* common x-axis in the Lower Panel, respectively for a healthy subject (left) and an HF subject (right). We can see the close relationship between the beginning of the linear part of the *V*´CO_2_-log*V*´_E_ slope (red line) with the Ɵ_L_, preceded by the smooth exponential increase (green curve). The transition is coincident with the increase in the ⩒*_E_*/⩒O_2’_ (blue circles) combined with the beginning of the ⩒*_E_*/⩒CO_2_ ventilatory isocapnic “buffering” period (green circles). The first vertical interrupted line-arrow points to the Ɵ_L_ transition, and the second vertical interrupted line-arrow points to the probable RCP correspondent projection. At the ⩒*_E_*/⩒CO_2_ ventilatory isocapnic “buffering” period, there is a proportional increase in both ⩒*_E_* and ⩒CO_2_, provoking a relatively flat ⩒*_E_*/⩒CO_2_ profile (between Ɵ_L_ and RCP). Abbreviations: RCP, Respiratory Compensation Point; Ɵ_L_, First VentilatoryThreshold.

We termed this slope coefficient (“**b**”) as the CO_2_ output constant rate (⩒CO_2_-log⩒_E_ slope), after excluding the non-linear segment. Similar to the OUES, this slope describes the rate of CO_2_ output during graded exercise, for each ten-fold increase in ⩒_E_. Subsequently, we ascribed the transition point from the exponential-to-linear part of the ⩒CO_2_-log⩒*_E_* slope to the beginning of the first ventilatory threshold (Ɵ_L_), after careful analysis of the entire sample. Thus, as we can see in Fig. 2, the beginning of the linear segment of the ⩒CO_2_-log⩒*_E_* response is approximately time-aligned with the Ɵ_L,_ both in a representative healthy subject, and in a representative HF participant. This is suggested by the time-alignment with the beginning of both (i) the increase in the ⩒_E_/⩒O_2_, and (ii) the beginning of the isocapnic ventilation “buffering” period for the ⩒*_E_***/**⩒CO_2_ equivalent. Thus, the ⩒CO_2_-log⩒_E_ slope could be calculated after the exclusion of the data before the Ɵ_L_.

### ⩒CO_2_-log⩒*_E_* max

**Fig. 3:**
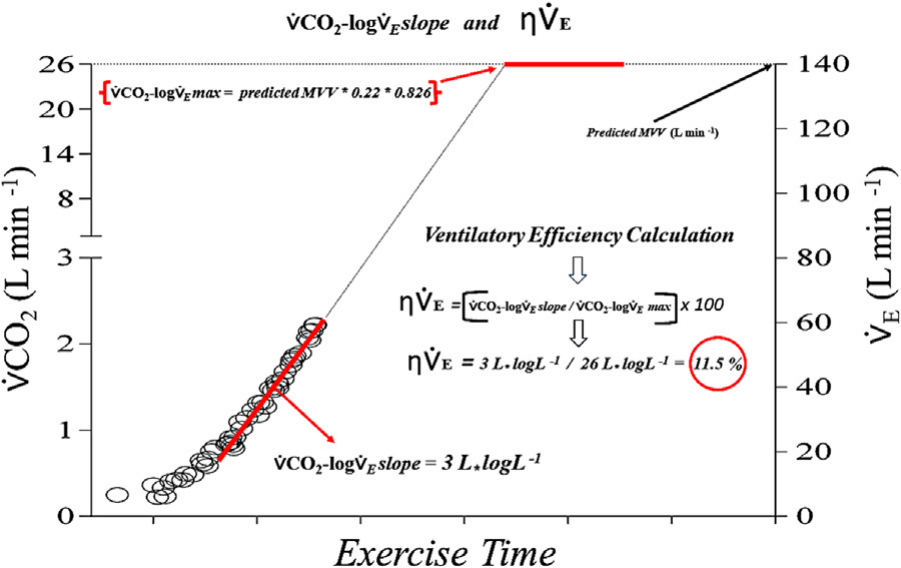
Example of ventilatory efficiency (η^⩒^_E_) calculation. In this three-axis plot, minute-ventilation was plotted without log scale and placed at the right for clarity. The linear regression of the linear part of the quadratic function is depicted as a red line, and the linear coefficient or actual CO_2_ output constant rate (⩒CO_2_-log⩒_E_ slope) is equal to 3 L_*_ logL_ˉ_¹ for this particular subject (below). The predicted MVV for this subject is 140 L/min and the maximum “assumed” CO_2_ output constant rate (⩒CO_2_-log⩒_E_ max) theoretically predicted for this subject would be 26 L_*_ logL_ˉ_¹ (see the text for elaboration). Thus, η^⩒^*_E_ = (*⩒CO_2_-log⩒_E_ slope / ⩒CO_2_-log⩒_E_ max) * 100 = 11,5 %. Reproduced with permission.

Furthermore, we assume a formalism for a theoretical maximal constant rate for clearing the CO_2_ from the lungs during exercise hyperpnea**,** or ⩒CO_2_-log⩒*_E_* max**_._** This theoretical ceiling could be achieved during incremental exercise if we assume a complete uptake of oxygen from the lungs, resulting in an expired fraction of oxygen (*F*E*O_2_*) of “0 %” and expired carbon dioxide fraction (*F*E*CO_2_*) of 22 % (for balanced nitrogen). While not physiologically achievable, this situation provides us with the theoretical upper limit for CO_2_ output. The maximum CO_2_ output attainable, or ⩒CO_2_-log⩒*_E_* max, can be practically calculated using the predicted MVV, as follows: ⩒CO_2_-log⩒_E_ max = MVV predicted _*_ 0.22 _*_ 0.826, where 0.826 is the ATPS to STPD conversion factor assuming ambient temperature and pressure corrections (see Fig. 3 for details). This hypothetical state is in accordance with the definition of “perfection” in physiology (Gans, C., 1991), or *“represents the best state that is conceivable. It would thus be equivalent to an efficiency of 100 %. It is obviously the abstract Platonic ideal…..”* [13].

### Ventilatory efficiency (η^⩒^_E_)

Subsequently, the ⩒CO_2_-log⩒_E_ max is compared to the ⩒CO_2_-log⩒_E_ slope, defining the true ventilatory efficiency index (η^⩒^_E_,%). Hence, we can measure the actual CO_2_ output constant rate during incremental exercise (⩒CO_2_-log⩒_E_ slope) and represent this as a proportion of the ⩒CO_2_-log⩒_E_ max, in order to obtain the η^⩒^_E_. This calculation would take the form:

(3) $${\eta^{\dot{\vee }}}_{E}=\left(\dot{\vee }CO_{2}-\text{log}{\dot{\vee }}_{E}\;\text{slope}/\dot{\vee }CO_{2}-\text{log}{\dot{\vee }}_{E}\;\text{max}\right)*100\;\left(\text{Figure}\;3\right)$$

The potential advantages of the η^⩒^_E_ compared to the ⩒CO_2_-log⩒_E_ slope are (i) fulfillment of the criteria for a true ventilatory efficiency index, as the actual rate of CO_2_ cleared from the lungs is compared to a theoretical ceiling for the same rate of CO_2_ (in “%”, as usually required for an efficiency index), and (ii) allowing the adjustment of the ⩒CO_2_-log⩒_E_ slope for age, sex, and/or body mass index, depending on reference equations for MVV. When reference equations for MVV are not available, a possible solution is to refer to the predicted FEV_1_ multiplied by a factor of 40. However, ideally, reference equations for η^⩒^_E_ would be the best approach.

## Ethics statements

Thirty-four subjects with Heart Failure (HF) according to the Brazilian Guideline for HF diagnosis criteria [17], and one healthy voluntary subject, were recruited and evaluated for cardiopulmonary exercise testing (CPET), consecutively, adhering to the human research medical and ethical standards outlined in the Declaration of Helsinki. Individuals voluntarily provided verbal and written informed consent prior to study participation. The study was approved by our Institution (CAAE 75,895,617.5.0000.0021).

## CRediT authorship contribution statement

**Paulo de Tarso Muller:** Conceptualization, Methodology, Data curation, Writing – original draft, Visualization, Investigation, Supervision, Writing – review & editing.

## Declaration of Competing Interest

The authors declare that they have no known competing financial interests or personal relationships that could have appeared to influence the work reported in this paper.

## Data Availability

No data was used for the research described in the article.
